# Fuzzy Multidimensional Model to Cluster Dengue Risk in Sri Lanka

**DOI:** 10.1155/2020/2420948

**Published:** 2020-11-04

**Authors:** I. T. S. Piyatilake, S. S. N. Perera

**Affiliations:** ^1^Department of Computational Mathematics, University of Moratuwa, Sri Lanka; ^2^Research & Development Center for Mathematical Modeling, Department of Mathematics, Faculty of Science, University of Colombo, Sri Lanka

## Abstract

Dengue is the world's rapidly transmitting mosquito-borne viral disease. It is mostly found in subtropical countries in the world. The annual number of global deaths caused by dengue fever is about 25,000. The Sri Lanka dengue situation is also not different to other countries. In the year 2019, dengue fever caused 120 deaths in Sri Lanka. Most of these deaths were reported from the main administrative district Colombo. Health authorities have to pay their attention to control this new situation. Therefore, identifying the hot spots in the country and implementing necessary actions to control the disease is an important task. This study aims to develop a clustering technique to identify the dengue hot spots in Sri Lanka. Suitable risk factors are identified using expert ideas and reviewing available literature. The weights are derived using Chang's extent method. These weights are used to prioritize the factors associated with dengue. Using the geometric mean, the interaction between the triggering variable and other variables is calculated. According to the interaction matrices, five dengue risk clusters are identified. It is found that high population movement in the area plays a dominant role to transmit the disease to other areas. Most of the districts in Sri Lanka will reach to moderate risk cluster in the year 2022.

## 1. Introduction

The World Health Organization (WHO) considered *dengue* as one of the world's top ten global health hazards in 2019 [1]. It is a mosquito-borne viral disease, and this virus belongs to the *Flaviviridae* family of viruses. Dengue virus appears as four different, but closely associated serotypes of viruses, namely, DENV-1, DENV-2, DENV-3, and DENV-4 [2]. Infected female mosquitoes of the categories *Aedes aegypti* and *Aedes albopictus* are found to be spreading the dengue virus. These viruses are getting to healthy human bodies by infected mosquito bites. If a human being recovers from one serotype of dengue, he will not have that type of dengue in future, but it does not grant the cross protection to other serotypes. A consecutive contamination by different serotypes is a risk factor for having severe forms of dengue, namely, Dengue Hemorrhagic Fever (DHF) and Dengue Shock Syndrome (DSS), and these two forms are considered as life-threatening stages of dengue [3]. High fever, headache, pain behind the eye, nausea, vomiting, muscle and joint aches, different types of rashes, bleeding from the nose and gums, and bruises and facial flushing are the symptoms of dengue fever. In addition, widespread bleeding, low blood pressure, and body organ such as liver and kidney failure can occur in DHF and DSS stages. Things such as used tires and food containers which can collect and store water and poor collecting and disposing of garbage in urban areas are some of the factors helping the breeding of the mosquitoes. Also, some rarely used water collecting methods such as water tanks, wells, and poor access to sanitation facilities help the breeding of the mosquitoes. Above all these, helpful climate effects for mosquito breeding process is significant.

WHO statistics indicate the risk of exposure to dengue is nearly half the world human population [1]. As an average, 390 million dengue cases were reported worldwide in every past year [1]. The annual number of global deaths caused by severe forms of dengue is about 20,000 [1]. Among the infected people of dengue, 90% of them are children and most of them are from Asian and Latin American countries. The global dengue treatment expenditure is about 8 billion dollars [4].

The situation in Sri Lanka is also not different to other countries. Colombo was the capital city and was the busiest industrial area of Sri Lanka in 1965 to 1966. The first dengue outbreak was reported in Colombo in the same period. Until 1989, the dengue virus progressed at a very slow rate and was identified as a sporadic disease. During the period 1989 to 1990, the number of recorded DHF cases was 206 and that is the first epidemic of dengue fever in Sri Lanka [5]. Thereafter, the numbers of dengue cases and deaths have abruptly increased. The year 2017 reported the largest outbreak of dengue in Sri Lanka with 186,101 cases and 440 deaths [6, 7]. While the Colombo district reported 18,186 cases, the Gampaha district reported 12,121 cases [8]. These two figures were the highest in the country. In recent decades, dengue becomes a leading public health problem in Sri Lanka, because the annual number of patients was increased by this disease. Not only that, it spreads to new areas due to infected travelers. For instance, in 1965, the dengue cases are initially found mostly in and around Colombo district, but they progressively spread to other districts and reached outbreak proportions in several main districts such as Gampaha, Kurunegala, Kalutara, Batticaloa, Kegalle, Ratnapura, Kandy, Matara, Galle, and Trincomalee districts in 2017 [9].

Still, vaccine has not been developed by scientist to prevent dengue attacks. Destroying mosquito breeding sites is of the utmost importance to control spreading dengue. Therefore, it is beneficial to identify the clusters with a similar risk of dengue transmission. This would facilitate the relevant authorities to implement the same control strategies in similar risk areas effectively. It also helps to allocate funds and resources efficiently based on the intensity of dengue risk. Therefore, this study is aimed at developing a multidimensional risk model to cluster the dengue hot spots in urban areas of Sri Lanka based on risk factors and predict the possible future hot spots by identifying the variation of appropriate factors.

Several studies focused to develop clustering algorithms to identify the area with a similar risk of dengue transmission. The study developed by Lowe et al. [10] proposed a Bayesian inference-based model which is used to identify the intensity of dengue risk in Brazil during the 2014 FIFA World Cup season. The factors altitude, population density, temperature, and rainfall were used to find the risk of dengue. The results of their study revealed that the risk of dengue was low in the selected cities for the football matches. A statistical analysis was performed by Vicente et al. [11] to detect the spatial variation of dengue temporal data in Brazil. They considered the relative risk of dengue, house index, population density, and income for their analysis. According to their study, there were 11 clusters in Vitória, Brazil. In the Sri Lankan context, there were a very limited number of studies carried out in this regard. Sumanasinghe et al. [12] developed a geostatistical risk model to cluster the high-risk dengue areas in Sri Lanka. The effect of rainfall and population density were considered for their study. They identified five risk areas using geographically weighted regression. Combining past dengue records with climate data, a new two cluster model was proposed by Sun et al. [13]. They used spatial-temporal clustering method, to investigate the dengue clusters in Sri Lanka from 2012 to 2016. All these Sri Lankan studies were based on only climate factors and population density. Therefore, it is worthwhile to build a multifactorial model considering social and climate factors to cluster the dengue risk areas. These social and climate factors are highly uncertain, and so, it is not possible to define the exact boundaries of those factors. In the present study, the combined effect of multifactors is used in an uncertain environment to find the dengue risk of a given area which was not discussed in the previous studies.

Defining multifactorial risk models is quite a sophisticated task due to the uncertainty nature of such factors. Fuzzy mathematics which was invented in 1965 by Zadeh [14] is one path to model the uncertainty situations. Therefore, fuzzy mathematical concepts are used to develop the problem. The model is constructed as a hierarchical process by including factors and their risk categories in two different levels. The analytical hierarchical process (AHP) is a mathematical tool which was invented by Saaty in 1970 to handle hierarchical processes. Using AHP, we can decompose the given decision problem into different subproblems which can easily handle. Due to the uncertainty of the factors in our problem, fuzzy analytical hierarchical process (FAHP) is used to construct the model. Finally, the dengue risk categories are constructed combining the results of FAHP and Haddon matrices.

The remaining sections of this paper are organized as follows.  Section 2 introduces mathematical approaches, including basic definitions of fuzzy theory, the FAHP technique, the concept of Haddon matrix, and geometric mean calculation which we used to build the risk model. The model construction and factor selection processes are presented in Section 3. The clustering algorithm, risk categories, and obtained results with discussion are presented in Section 4. Finally, the conclusion and remarks can be found.

## 2. Theoretical Background

In this section, we will look at some mathematical theories including commonly used definitions in fuzzy theory, Chang's extent analysis method, geometric mean, and Haddon matrix. These theories are gathered from the references [15–21].

### 2.1. Basic Definitions in Fuzzy Theory

**Definition 1 .**

A fuzzy set, *A*, in a universe of discourse, *U*, is a function of the form

(1) $$\begin{array}{c} f_{A}:U\to \left[0,1\right]. \end{array}$$

The function *f*_*A*_ is called the membership function of *A*, and for any *x* in *U*, *f*_*A*_(*x*) in [0, 1] represents the grade of membership of *x* in *A*.

**Definition 2 (triangular fuzzy number).**

A triangular fuzzy number (TFN), *A*, can be defined by a triplet (*l*, *m*, *u*), where *l*, *m*, *u* represent the smallest possible value, the most promising value, and the largest possible value of an event, respectively. This representation is interpreted using the membership function as follows:

(2) $$\begin{array}{c} f_{A}\left(x\right)=\left(\begin{array}{cc} \frac{x-l}{m-l}, & \text{if }x\in \left[l,m\right]\\ \frac{u-x}{u-m}, & \text{if }x\in \left[m,u\right]\\ 0, & \text{otherwise}. \end{array}\right. \end{array}$$

**Definition 3 (basic operations).**

Assume that *A* and *B* are two triangular fuzzy numbers with *A* = (*l*_1_, *m*_1_, *u*_1_) and *B* = (*l*_2_, *m*_2_, *u*_2_). The basic operations are:

1. Addition

(3) $$\begin{array}{c} A⊕B=\left(l_{1}+l_{2},m_{1}+m_{2},u_{1}+u_{2}\right). \end{array}$$

(2) Multiplication

(4) $$\begin{array}{c} A⊗B=\left(l_{1}l_{2},m_{1}m_{2},u_{1}u_{2}\right). \end{array}$$

(3) Inverse

(5) $$\begin{array}{c} A^{-1}\approx \left(\frac{1}{u_{1}},\frac{1}{m_{1}},\frac{1}{l_{1}}\right). \end{array}$$

### 2.2. Fuzzy Pair Wise Comparison Matrix

Let $\tilde{A}$ represent a fuzzified reciprocal *n* × *n* judgment matrix containing all pairwise comparisons ${\tilde{a}}_{ij}$ between elements *i* and *j* for all *i*, *j* ∈ 1, 2, 3, ⋯, *n*.

(6) $$\begin{array}{c} \tilde{A}=\begin{array}{c} c_{1}\\ c_{2}\\ \vdots \\ c_{n} \end{array}\left[\begin{array}{cccc} c_{1} & c_{2} & \cdots & c_{n}\\ \left(1,1,1\right) & {\tilde{a}}_{12} & \cdots & {\tilde{a}}_{1n}\\ {\tilde{a}}_{21} & \left(1,1,1\right) & \cdots & {\tilde{a}}_{2n}\\ \vdots & \vdots & \ddots & \vdots \\ {\tilde{a}}_{n1} & {\tilde{a}}_{n2} & \cdots & \left(1,1,1\right) \end{array}\right], \end{array}$$

where ${\tilde{a}}_{ij}=\left(1,1,1\right)\text{: }\forall i=j$, ${\tilde{a}}_{ji}={\tilde{a}}_{ij}^{-1}$, *n* is the criteria number to be evaluated, *c*_*i*_ is the *i*th criteria, ${\tilde{a}}_{ij}$ is the importance of the *i*th criteria according to the *j*th criteria, and all ${\tilde{a}}_{ij}$ are triangular fuzzy numbers ${\tilde{a}}_{ij}=\left(l_{ij},m_{ij},u_{ij}\right)$.

### 2.3. Linguistic Variable

**Definition 4 (linguistic variable).**

A linguistic variable [14] is characterized by a quintuple $\left(x,T\left(x\right),U,G,\tilde{M}\right)$, in which *x* is the name of the variable, *T*(*x*) denotes the term set of *x*, that is, the set of names of linguistic values of *x*. Each of these values is a fuzzy variable, denoted generically by *X* and ranging over a universe of discourse *U*, which is associated with the base variable *u*; *G* is a syntactic rule (which usually has the form of a grammar) for generating the name, *X*, of values of *x*. *M* is a semantic rule for associating with each *X* its meaning. Here, $\tilde{M}\left(X\right)$ is a fuzzy subset of *U*. A particular *X*, that is, a name generated by *G*, is called a term.

For the analysis purposes, the expert judgments regarding variables are processed using linguistic variables. Traditionally, we represent these linguistic variables using numerical scales. Commonly used numerical scales are 1 to 3, 1 to 5, 1 to 7, and 1 to 9. As we know, in the real scenario, these expert judgments are uncertain. Therefore, in this study, these uncertain ideas are analyzed using triangular fuzzy numbers. The linguistic scale and their triangular fuzzy representation are shown in Table 1.

### 2.4. Chang's Extent Analysis Method

Let *X* = {*x*_1_, *x*_2_, *x*_3_, ⋯, *x*_*n*_} be an object set and *G* = {*g*_1_, *g*_2_, *g*_3_, ⋯, *g*_*n*_} be a goal set. Then, each object is taken and the extent analysis for each goal is performed, respectively. There are *m* extent analysis values for each object, and the values are *M*_*g*_*i*__^1^, *M*_*g*_*i*__^2^, ⋯, *M*_*g*_*i*__^*m*^, *i* = 1, 2, ⋯, *n* where *M*_*g*_*i*__^*j*^(*j* = 1, 2, ⋯, *m*) are TFNs. The steps of Chang's extent analysis method are as follows:

**Step 1 .**

The value of fuzzy synthetic extent with respect to the *i*th object is

(7) $$\begin{array}{c} s_{i}=\overset{m}{\underset{j=1}{\sum }}\;\;M_{g_{i}}^{j}⊗{\left[\overset{n}{\underset{i=1}{\sum }}\;\;\overset{m}{\underset{j=1}{\sum }}\;\;M_{g_{i}}^{j}\right]}^{-1}. \end{array}$$

The value of ∑_*j*=1_^*m*^  *M*_*g*_*i*__^*j*^ is obtained using the “fuzzy addition operation” of *m* extent analysis values for a particular matrix, and the value [∑_*i*=1_^*n*^  ∑_*j*=1_^*m*^  *M*_*g*_*i*__^*j*^]^−1^ is obtained using the “fuzzy addition operation” of *M*_*g*_*i*__^*j*^(*j* = 1, 2, ⋯, *m*) values.

**Step 2 .**

The degree of possibility of two triangular fuzzy numbers *M*_2_ = (*l*_2_, *m*_2_, *u*_2_) ≥ *M*_1_ = (*l*_1_, *m*_1_, *u*_1_) can be expressed as follows:

(8) $$\begin{array}{c} V\left(M_{2}\geq M_{1}\right)=\left(\begin{array}{cc} 1 & \text{if }m_{2}\geq m_{1}\\ 0 & \text{if }l_{1}\geq u_{2}\\ \frac{l_{1}-u_{2}}{\left(m_{2}-u_{2}\right)-\left(m_{1}-l_{1}\right)}, & \text{otherwise}. \end{array}\right. \end{array}$$

According to the values of *V*(*M*_1_ ≥ *M*_2_) and *V*(*M*_2_ ≥ *M*_1_), two triangular fuzzy numbers can be compared.

**Step 3 .**

**Figure 1:**
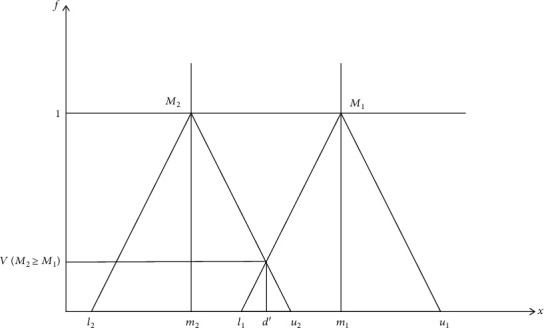
The intersection of *M*_1_ and *M*_2_.

Assume that

(9) $$\begin{array}{c} d^{\prime }\left(A_{i}\right)=\min V\left(S_{i}\geq S_{k}\right), \end{array}$$

for *k* = 1, 2, ⋯, *n*; *k* ≠ *i*, where *d*′ is the ordinate of the highest intersection point *D* between *μ*_*M*_1__ and *μ*_*M*_2__, and it is shown in Figure 1.

**Step 4 .**

Then, the weight vector is given by

(10) $$\begin{array}{c} W^{\prime }={\left(d^{\prime }\left(A_{1}\right),d^{\prime }\left(A_{2}\right),\cdots ,d^{\prime }\left(A_{n}\right)\right)}^{T}, \end{array}$$

where *A*_*i*_, (*i* = 1, 2, ⋯, *n*) are *n* elements.

**Step 5 .**

Normalizing (10) can obtain the normalized weight vector

(11) $$\begin{array}{c} W={\left(d\left(A_{1}\right),d\left(A_{2}\right),\cdots ,d\left(A_{n}\right)\right)}^{T}, \end{array}$$

where *d*(*A*_*i*_) = (*d*′(*A*_*i*_))/(∑_*i*=1_^*n*^  *d*′(*A*_1_)). Now, *W* is a nonfuzzy number.

### 2.5. Haddon Matrix

The concept of the Haddon matrix [22] was invented by William Haddon in 1970. This was first introduced as a tool to recognize the risk factors associated with injury occurrences. This conceptual framework facilitates users to think about injury prevention activities and their association with factors in three stages: before injury, injury, and after injury. The cells of this matrix provide users different approaches to prevent the injury.

### 2.6. Geometric Mean

Geometric mean (GM) [23] is used to measure the central location of a given data set. For a given set of positive random variables {*x*_*i*_}_*i*=1_^*N*^, we can define GM value as follows:

(12) $$\begin{array}{c} \text{GM}=\sqrt[{N}]{\overset{N}{\underset{i=1}{\prod }}\;\;x_{i}}={\left(\overset{N}{\underset{i=1}{\prod }}\;\;x_{i}\right)}^{\frac{1}{N}}, \end{array}$$

where *N* is the population (or sample) size. For the zero and negative random variables, we can define GM value considering the following three cases.

**Case 1 .**

Given *N* is an odd number and all the random variables {*x*_*i*_}_*i*=1_^*N*^ are less than 0, in this situation, GM value is given by

(13) $$\begin{array}{c} \text{GM}=\sqrt[{N}]{\overset{N}{\underset{i=1}{\prod }}\;\;\left(-x_{i}\right)}=-\sqrt[{N}]{\overset{N}{\underset{i=1}{\prod }}\;\;\left|x_{i}\right|}. \end{array}$$

**Case 2 .**

Given *N*_1_ is the number of a negative random variable in the given set of data and contains both positive and negative values, then the GM value is given by

(14) $$\begin{array}{c} \text{GM}=\sqrt[{N}]{\overset{N}{\underset{i=1}{\prod }}\;\;x_{i}}=\sqrt[{N}]{\overset{N_{1}}{\underset{i=1}{\prod }}\;\;x_{i}^{-}\overset{N}{\underset{i=N_{1}+1}{\prod }}\;\;x_{i}^{+}}=\sqrt[{N}]{\overset{N_{1}}{\underset{i=1}{\prod }}\;\;x_{i}^{-}}\sqrt[{N}]{\overset{N}{\underset{i=N_{1}+1}{\prod }}\;\;x_{i}^{+}}. \end{array}$$

According to (14), the total GM value is the multiplication of GM of negative values and GM of positive values. Therefore, this total GM value can be called as bigeometrical. In this case, the total GM can be calculated as a weighted average, and it is given by,

(15) $$\begin{array}{c} \text{GM}=W_{1}G_{-}+W_{2}G_{+}, \end{array}$$

where $G_{-}=\sqrt[{N}]{\prod_{i=1}^{N_{1}}\;\;x_{i}^{-}}$, $G_{+}=\sqrt[{N}]{\prod_{i=N_{1}+1}^{N}\;\;x_{i}^{+}}$, *W*_1_ = *N*_1_/*N*, and *W*_2_ = *N*_2_/*N*. Here, *N*_2_ is the number of positive values in the set.

**Case 3 .**

Given some of the random variable values are equal to 0, in this situation, we should consider about three different GM values to compute the overall GM. Therefore, this GM value is called as trigeometrical. In here, the overall GM value is given by

(16) $$\begin{array}{c} \text{GM}=W_{1}G_{-}+W_{2}G_{+}+W_{3}G_{0}, \end{array}$$

where $G_{-}=\sqrt[{N}]{\prod_{i=1}^{N_{1}}\;\;x_{i}^{-}}$, $G_{+}=\sqrt[{N}]{\prod_{i=N_{1}+1}^{N_{2}}\;\;x_{i}^{+}}$, *G*_0_ = 0, *W*_1_ = *N*_1_/*N*, *W*_2_ = *N*_2_/*N*, and *W*_3_ = *N*_3_/*N*. Here, *N*_2_ is the number of positive values in the set, *N*_3_ is the number of zero values in the set, and *N* = *N*_1_ + *N*_2_ + *N*_3_.

## 3. Methodology

### 3.1. Selection of Predictive Factors

**Figure 2:**
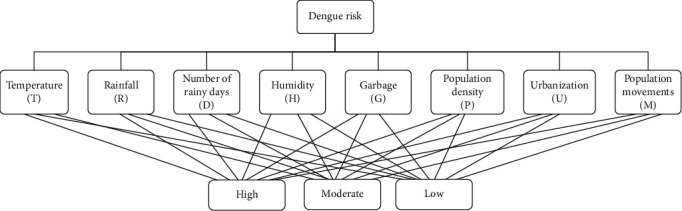
The proposed hierarchical model.

After reviewing the facts of the national and international sources regarding the dengue transmission, eight different predictive factors are identified. They are the average value of daily temperature (°C), average value of daily rainfall (mm), average number of rainy days per week, average value of daily humidity (%), average daily collection of garbage (Mt/Day), population density (persons/km^2^), percentage of urbanization, and number of population movements to the target area. By considering the impact of these factors on dengue severity, they are further subdivided into three risk levels, namely, high, moderate, and low. Expert ideas and literature are used for this purpose [21, 24–26]. The selected factors with their subcategories are given in Table 2. Using all the above facts, the proposed hierarchical model is presented in Figure 2.

### 3.2. Constructing Multidimensional Dengue Risk Clusters

**Figure 3:**
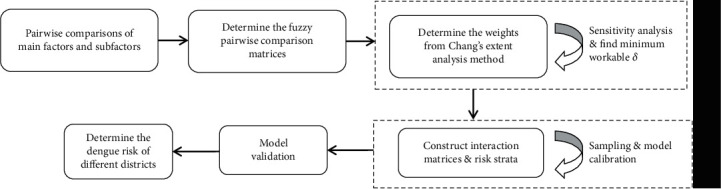
The framework for dengue risk model development.

We pursue the steps which are presented in Figure 3 to construct the risk clusters. Using expert opinions in the field, the subfactors are compared and constructed the fuzzy pair-wise comparisons matrices. The weights of the factors are derived using Chang's extent analysis method. According to the derived weights, the factor with the highest numerical value is defined as the trigger variable (*Ω*) in the context of dengue transmission. Then, the interaction between the variable *Ω* and the remaining seven factors (*Φ*; *i* = 1, 2, ⋯, 7) are calculated. These calculated interaction values are stored in seven 3 × 3 matrices as in Table 3 [21, 24]. Finally, these seven matrices are combined using the GM values as in Table 4.

According to the output of Table 4 and using the concept of the Haddon matrices, three risk clusters can be defined as follows:

1. *Low-risk cluster*. The value of *R*_*L*,*L*_ is the lower limit, and the value of *R*_*M*,*L*_ is the upper limit of this cluster.
2. *Moderate risk cluster*. The value of *R*_*M*,*H*_ is the lower limit, and the value of *R*_*M*,*L*_ is the upper limit of this cluster.
3. *High-risk cluster*. The value of *R*_*M*,*H*_ is the lower limit, and the value of *R*_*H*,*H*_ is the upper limit of this cluster.

To find the risk clusters of districts membership values of predictive factors are aggregated using GM. MATLAB and R statistical softwares are used to carry out the simulations.

## 4. Results

### 4.1. Algorithm

**Step 6 .**

Compare the predictive factors using expert ideas.

With the aid of expert ideas, pairwise comparison matrices as in Table S1 to Table S9 (see Supplementary material S1) are created.

**Step 7 .**

Prioritize the predictive factors.

Synthetic extent value of each factor is derived using (7) and the results in Table S1 (see Supplementary material S1). These derived values are given as follows:

**Table:** 

*S*_*T*_ = (0.0644,0.1062,0.1713)	*S*_*R*_ = (0.0577,0.1045,0.1798)
*S*_*D*_ = (0.0601,0.1058,0.1854)	*S*_*H*_ = (0.0500,0.0794,0.1320)
*S*_*G*_ = (0.1023,0.1749,0.2949)	*S*_*P*_ = (0.0527,0.0859,0.1545)
*S*_*U*_ = (0.0953,0.1554,0.2584)	*S*_*M*_ = (0.1148,0.1878,0.2978)

Then, to find the degrees of possibilities above, the values are compared according to the condition given in (8). The results are given as follows:

**Table:** 

*V*(*S*_*T*_ ≥ *S*_*R*_) = 1	*V*(*S*_*R*_ ≥ *S*_*T*_) = 0.9852	*V*(*S*_*D*_ ≥ *S*_*T*_) = 0.9964
*V*(*S*_*T*_ ≥ *S*_*D*_) = 1	*V*(*S*_*R*_ ≥ *S*_*D*_) = 0.9893	*V*(*S*_*D*_ ≥ *S*_*R*_) = 1
*V*(*S*_*T*_ ≥ *S*_*H*_) = 1	*V*(*S*_*R*_ ≥ *S*_*H*_) = 1	*V*(*S*_*D*_ ≥ *S*_*H*_) = 1
*V*(*S*_*T*_ ≥ *S*_*G*_) = 0.5013	*V*(*S*_*R*_ ≥ *S*_*G*_) = 0.5238	*V*(*S*_*D*_ ≥ *S*_*G*_) = 0.5459
*V*(*S*_*T*_ ≥ *S*_*P*_) = 1	*V*(*S*_*R*_ ≥ *S*_*P*_) = 1	*V*(*S*_*D*_ ≥ *S*_*P*_) = 1
*V*(*S*_*T*_ ≥ *S*_*U*_) = 0.6071	* V*(*S*_*R*_ ≥ *S*_*U*_) = 0.6238	* V*(*S*_*D*_ ≥ *S*_*U*_) = 0.6447
*V*(*S*_*T*_ ≥ *S*_*M*_) = 0.4095	* V*(*S*_*R*_ ≥ *S*_*M*_) = 0.4382	* V*(*S*_*D*_ ≥ *S*_*M*_) = 0.4626
*V*(*S*_*H*_ ≥ *S*_*T*_) = 0.7163	*V*(*S*_*G*_ ≥ *S*_*T*_) = 1	*V*(*S*_*P*_ ≥ *S*_*T*_) = 0.8161
*V*(*S*_*H*_ ≥ *S*_*R*_) = 0.7479	*V*(*S*_*G*_ ≥ *S*_*R*_) = 1	*V*(*S*_*P*_ ≥ *S*_*R*_) = 0.8390
*V*(*S*_*H*_ ≥ *S*_*D*_) = 0.7320	*V*(*S*_*G*_ ≥ *S*_*D*_) = 1	*V*(*S*_*P*_ ≥ *S*_*D*_) = 0.8262
*V*(*S*_*H*_ ≥ *S*_*G*_) = 0.2372	*V*(*S*_*G*_ ≥ *S*_*H*_) = 1	*V*(*S*_*P*_ ≥ *S*_*H*_) = 1
*V*(*S*_*H*_ ≥ *S*_*P*_) = 0.9245	*V*(*S*_*G*_ ≥ *S*_*P*_) = 1	*V*(*S*_*P*_ ≥ *S*_*G*_) = 0.3696
*V*(*S*_*H*_ ≥ *S*_*U*_) = 0.3258	*V*(*S*_*G*_ ≥ *S*_*U*_) = 1	*V*(*S*_*P*_ ≥ *S*_*U*_) = 0.4599
*V*(*S*_*H*_ ≥ *S*_*M*_) = 0.1374	* V*(*S*_*G*_ ≥ *S*_*M*_) = 0.9329	* V*(*S*_*P*_ ≥ *S*_*M*_) = 0.2805
*V*(*S*_*U*_ ≥ *S*_*T*_) = 1	*V*(*S*_*M*_ ≥ *S*_*T*_) = 1	
*V*(*S*_*U*_ ≥ *S*_*R*_) = 1	*V*(*S*_*M*_ ≥ *S*_*R*_) = 1	
*V*(*S*_*U*_ ≥ *S*_*D*_) = 1	*V*(*S*_*M*_ ≥ *S*_*D*_) = 1	
*V*(*S*_*U*_ ≥ *S*_*H*_) = 1	*V*(*S*_*M*_ ≥ *S*_*H*_) = 1	
*V*(*S*_*U*_ ≥ *S*_*G*_) = 0.8893	*V*(*S*_*M*_ ≥ *S*_*G*_) = 1	
*V*(*S*_*U*_ ≥ *S*_*P*_) = 1	*V*(*S*_*M*_ ≥ *S*_*P*_) = 1	
*V*(*S*_*U*_ ≥ *S*_*M*_) = 0.8161	*V*(*S*_*U*_ ≥ *S*_*T*_) = 1

Then, using (10), the minimum degree of possibilities is calculated. Normalizing these values according to (11), the final weights of the factors are derived. Then, the final weight vector of the predictive factors is

(17) $$\begin{array}{c} W=\left(0.0915,0.0979,0.1033,0.0307,0.2084,0.0627,0.1823,0.2234\right). \end{array}$$

**Figure 4:**
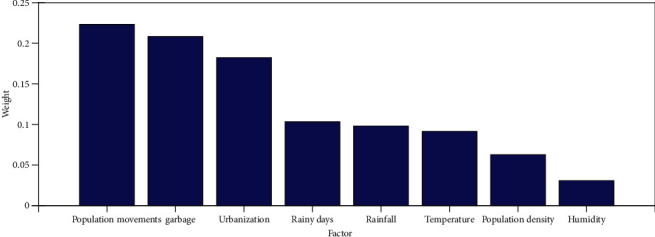
Prioritized weights of the predictive factors.

Similarly, we can derive the weights of the sublevel of the factors, and these values are given in Table 5. Finally, the derived weights are used to prioritize the predictive factors as shown in Figure 4. According to the output of Figure 4, the population movements in the area have the highest weight. Therefore, the variable population movements in the area are selected as *Ω*.

**Step 8 .**

Justification of selection of *δ*.

**Figure 5:**
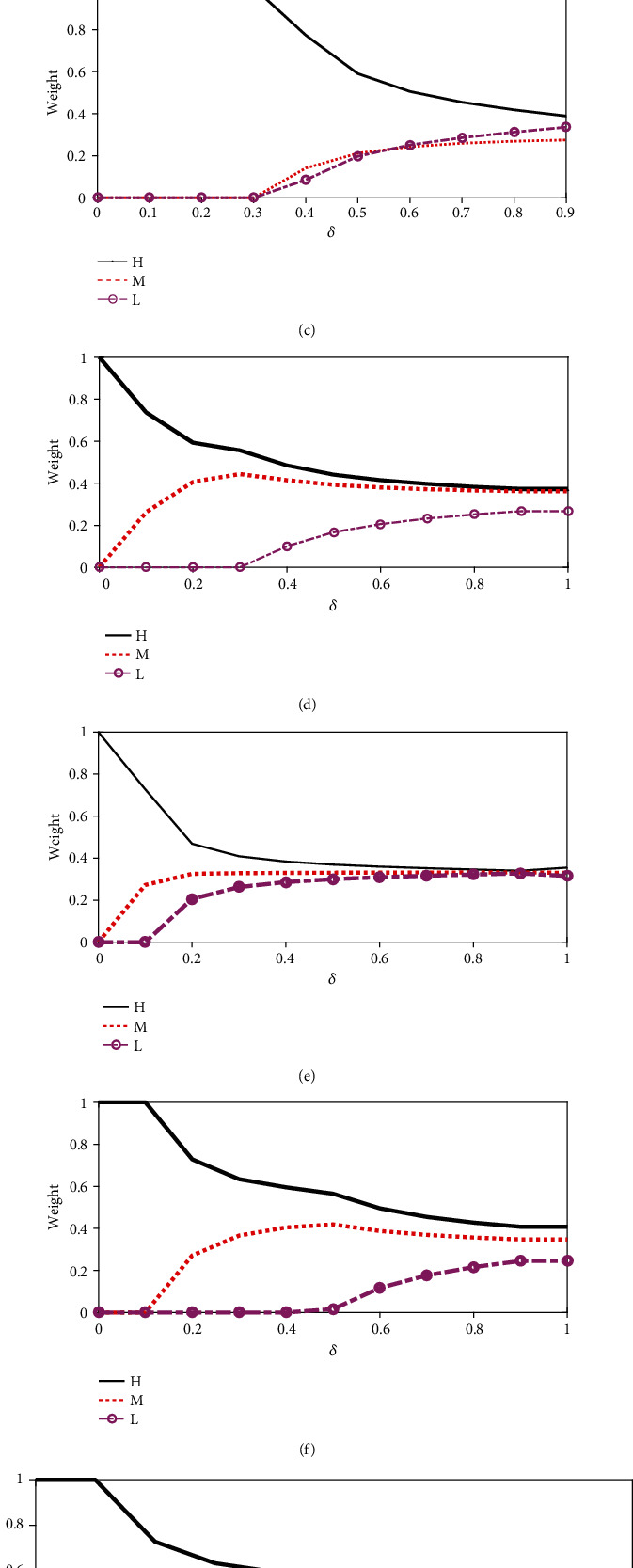
Sensitivity of the weights of factors for different values of *δ*: (a) all predictive factors; (b) temperature; (c) rainfall; (d) number of rainy days; (e) humidity; (f) garbage (g); population density; (h) urbanization.

In this step, we carry out a sensitivity analysis. This is to identify the impact of *δ* to the weights of the factors. In order to identify this, we carry out simulations for different values of *δ*, and these results are shown in Figure 5. According to the simulated results, we can observe that some of the weights are equal to 0 for some values of *δ*. Therefore, we must select a value for *δ* which gives a weight value for all the factors. For this study, we select this *δ* value as 0.5.

**Step 9 .**

Define dengue risk clusters.

The multiplicative interaction between each predictive factor with the subcategories of population movements is calculated using the concept explained in Table 3. These results are shown in Table S10 to Table S16 (see Supplementary material S2).

As explained in Table 4, the cells of Table S10 to Table S16 (see Supplementary material S2) combined using GM. The generated results are in Table 6. The proposed risk clusters and their boundaries based on levels of dengue risk with the proposed color scheme are shown in Table 7.

**Step 10 .**

Model calibration.

**Figure 6:**
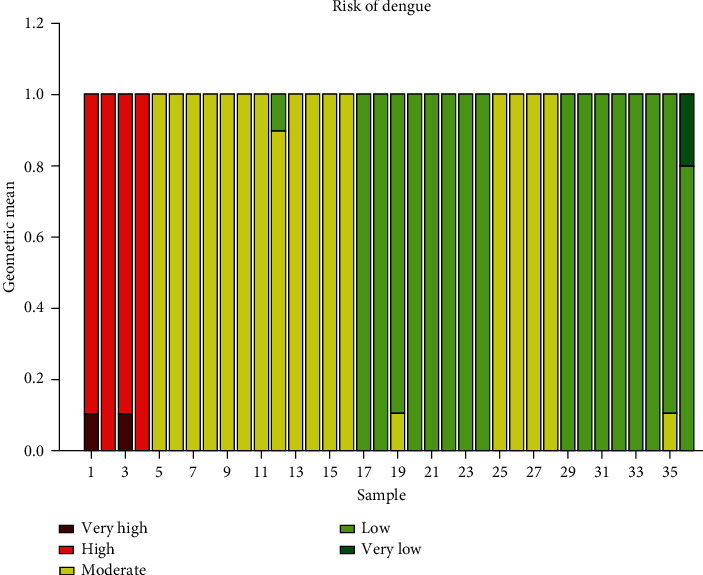
Model calibration with different samples.

The random sampling technique is used to calibrate the proposed model [21, 24]. For each sublevel of the trigger variable, 36 random samples are generated. To generate these samples, other variables are kept in three different probability levels. These probability levels are 80%, 50%, and 20%. For example, think that the trigger variable is in its high-risk sublevel. Then, one variable is selected with 80% probability and the remaining six variables selected with its complementary probability of 20%. Likewise, 10 repeated samples are generated randomly for this selection. Then, using the GM value, the risk cluster of each sample is generated. Then, the number of clusters with similar results are counted. Similarly, random samples are generated for other sublevels of the trigger variable, and the results are given in Figure 6.

As per Figure 6, we can identify the following three population movement clusters.

1. Cluster with high population movements

In Figure 6, the first twelve samples are generated with high population movements. According to Figure 6, we can see maroon, red, and yellow bars appear in this region. That means, when there are high population movements, the dengue risk belong to very high, high, and moderate dengue clusters.

(2) Cluster with moderate population movement

In Figure 6, the second set of twelve samples are generated with moderate population movements. According to Figure 6, we can see yellow and light green bars appear in this region. That means, when there are moderate population movements, the dengue risk belongs to the moderate and low dengue clusters.

(3) Cluster with low population movement

In Figure 6, the last set of twelve samples are generated with low population movements. According to Figure 6, we can see yellow, light green, and green bars appear in this region. That means, when there are low population movements, the dengue risk belongs to moderate, low, and very low dengue clusters.

### 4.2. Model Validation

**Figure 7:**
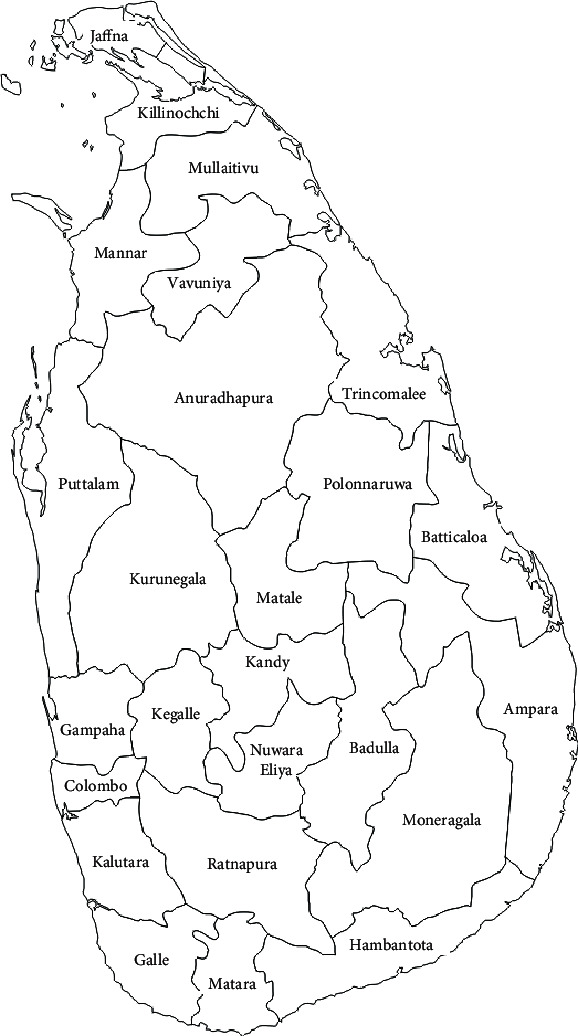
A map of the districts in Sri Lanka.

Twenty-two districts in Sri Lanka are selected for the model validation. The selected districts are Ampara, Anuradhapura, Badulla, Batticaloa, Colombo, Galle, Gampaha, Jaffna, Kalutara, Kandy, Kegalle, Kilinochchi, Kurunegala, Hambantota, Mannar, Matara, Mullaitivu, Nuwara Eliya, Puttalam, Ratnapura, Trincomalee, and Vavuniya. These districts are shown in Figure 7. Three districts, namely, Matale, Moneragala, and Polonnaruwa, are neglected from the study due to the unavailability of data. The risk factor details of these districts are collected from the Department of Census and Statistics, Epidemiology Unit of Health Ministry, Department of Meteorology, and Central Bank of Sri Lanka websites.

Using the proposed model, GM value of each district is generated. The obtained results and risk categories of districts are given in Table 8. Then, the generated risk categories are compared with the percentage relative value of dengue cases considering the year 2017 and the risk clusters announced by the International Federation of Red Cross and Red Crescent Societies [9]. According to the results, the model accuracy level is 72.73%.

The results revealed that the Colombo districts have the highest risk in terms of dengue in the year 2017, and it belongs to the high-risk cluster. Since Colombo is the main administrative district in Sri Lanka, main industries, schools, and universities are situated in this area. It also a highly dense area in Sri Lanka. Due to these reasons, there is a high dengue transmission in the Colombo district. Gampaha, Kalutara, Kurunegala, Kandy, Batticaloa, Jaffna, Ampara, Mannar, Kilinochchi, and Mullaitivu belong to moderate-risk cluster in the context of dengue transmission. The districts in the low-risk cluster are Anuradhapura, Badulla, Galle, Hambantota, Kegalle, Matara, Nuwara Eliya, Puttalam, Ratnapura, Trincomalee, and Vavuniya.

### 4.3. Capture the Future Risk Clusters of Dengue in Sri Lanka

**Figure 8:**
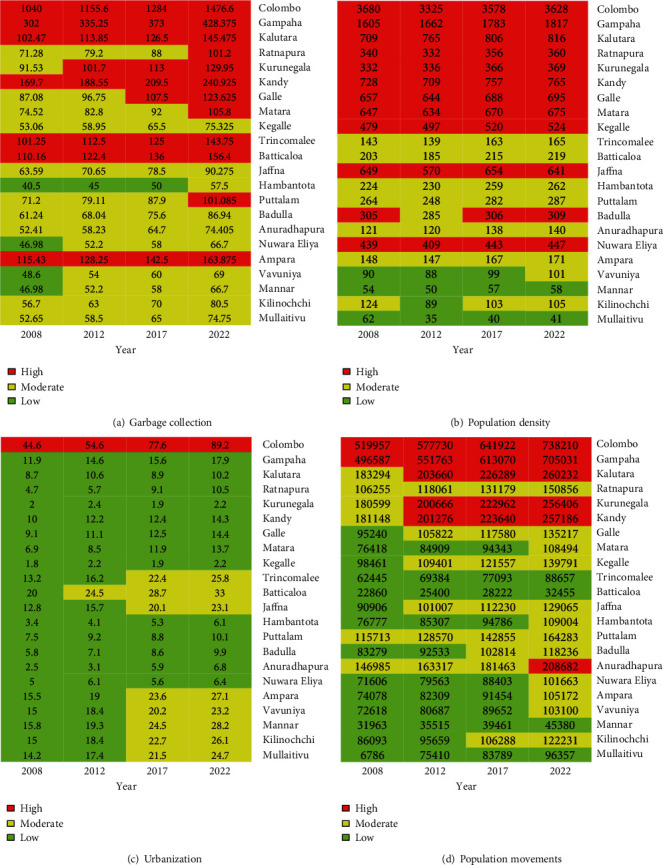
Heat maps of predictive factors.

**Figure 9:**
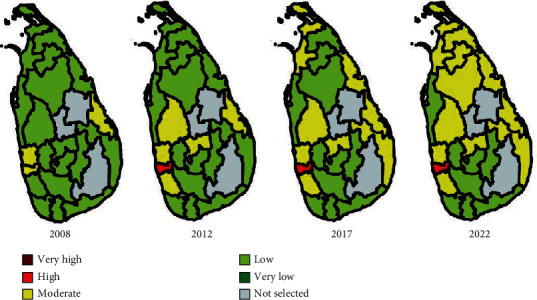
Dengue risk maps for the years 2008, 2012, 2017, and 2022.

It is important to predict the future dengue risk cluster in districts. In order to recognize the increasing trend of dengue clusters, 2008, 2012, and 2017 epidemic years are considered. The dengue risk prediction is carried out considering the year 2022. In order to simulate the results, we assumed that there is no change in the climate factors, the garbage increasing rate is 10%, the urbanization rate is 10%, and the population movements increase in selected areas by 10%. The annual population growth rates of the selected districts are obtained from the Department of Census and Statistics [27].  Figure 8 shows the heat maps generated for dengue risk factors.  Figure 9 shows the heat map generated to visualize the past and future risk clusters of different Sri Lankan districts.

## 5. Discussion

This study presents the multifactorial model to determine the dengue risk of cities in an uncertain environment. Eight different social and climate factors are considered for the model development process. The developed mathematical model was applied to Sri Lankan districts. According to the results of the present study, we identified the population movements in the area as a significant factor to increase the dengue risk. This result agrees with the findings of the literature [28, 29]. The model predicted the dengue risk in selected districts with 72.73% accuracy level. Out of 22 districts, 16 district risk clusters have been predicted accurately by the developed model. Correctly predicted districts are Anuradhapura, Badulla, Batticaloa, Colombo, Gampaha, Hambantota, Jaffna, Kalutara, Kandy, Kilinochchi, Kurunegala, Mannar, Mullaitivu, Nuwara Eliya, Puttalam, and Vavuniya.

Variables such as garbage collection, urbanization percentage, and population movements considered in this research are novel factors for dengue risk prediction models because the literature contains the results considering only the climate factors and population density. Most of the models in the literature were developed considering the statistical concepts like logistic regression [30, 31]. However, when we are dealing with more factors, there is an uncertainty of the model. In order to handle this uncertainty, a fuzzy theory-based mathematical model is developed. Also, in the proposed model, dengue risk clusters are defined by intervals. Hence, small changes in factors do not show a drastic change in the results. Further, this study considers the effect of multifactors and their interaction to predict the dengue risk. This also helps to increase the predictive effect of the present study compared to the existing studies.

## 6. Conclusions

In this research, a multidimensional risk-based model was introduced to cluster the dengue hot spots in Sri Lanka. The model incorporates the social and climate factors including the eight different variables which can be easily assessed and analyzed. Since these variables are highly uncertain, FAHP was used to prioritize the variables. The interactions of the variables were calculated using GM. Considering the concept of Haddon matrices, five dengue risk clusters were identified. High population movement to target district plays a dominant role to transmit the disease.

Due to the limited resources, we selected only a few risk factors of dengue for model construction. Therefore, it is necessary to include social variables such as school density, factory density, household density, conditions of houses, and infrastructure facilities in the area to the model, considering more number of factors will help to recognize the dengue transmission risk in districts more accurately. Here, we measure the interaction between two variables. But there may be multidimensional interaction between the dengue risk factors. It would be necessary to incorporate these multifactor interactions to the model in future studies. In this study, the obtained weights do not depend on time. However, in the real scenario, the selected factors such as rainfall, temperature, and humidity heavily depend on time. Hence, in the future studies, we can consider the time-dependent FAHP model to prioritize the variable. Note that the developed risk clustering algorithm can be used to identify future dengue hotspots in the country. This will facilitate the government and relevant authorities to develop control strategies. It also provides information to people to make correct decisions when they travel places with a high risk of dengue.

## Figures and Tables

**Table 1:** Linguistic variables used in the model construction and their triangular fuzzy number representation.

Linguistic term	Triangular fuzzy scale	Fuzzy scale for *δ* = 0.5
Absolutely more important	(3 − *δ*, 3, 3 + *δ*)	(5/2,3,7/2)
Very strongly more important	(5/2 − *δ*, 5/2, 5/2 + *δ*)	(2,5/2,3)
Strongly more important	(2 − *δ*, 2, 2 + *δ*)	(3/2,2,5/2)
Weakly more important	(3/2 − *δ*, 3/2, 3/2 + *δ*)	(1,3/2,2)
Equally important	(1 − *δ*, 1, 1 + *δ*)	(1/2,1,3/2)
Just equal	(1,1,1)	(1,1,1)

**Table 2:** Selected risk factors with their subcategories.

Risk factor	Risk level		
	High	Moderate	Low
T	30°C < *T* < 34°C	16°C ≤ *T* ≤ 30°C or 34°C ≤ *T* ≤ 37°C	*T* < 16°C or *T* > 37°C
R	10 mm < *R* < 30 mm	5 mm ≤ *R* ≤ 10 mm or 30 mm ≤ *R* ≤ 55 mm	*R* < 5 mm or *R* > 55 mm
D	*D* > 4	2 ≤ *D* ≤ 4	*D* < 2
H	*H* > 75	50 ≤ *H* ≤ 75	*H* < 50
G	*G* > 100	50 ≤ *G* ≤ 100	*G* < 50
P	*P* > 300	100 ≤ *P* ≤ 300	*P* < 100
U	*U* > 40	20 ≤ *U* ≤ 40	*U* < 20
M	*M* > 200, 000	100, 000 ≤ *M* ≤ 200, 000	*M* < 100, 000

**Table 3:** Matrix to find the interaction between the trigger variable with the other variables [21].

Risk level of variables		Risk level of trigger variable (*Ω*)		
		High	Moderate	Low
*Φ*; *i* = 1, 2, ⋯, 7	High	*Ω*_High_ × *Φ*_High_	*Ω*_Moderate_ × *Φ*_High_	*Ω*_Low_ × *Φ*_High_
	Moderate	*Ω*_High_ × *Φ*_Moderate_	*Ω*_Moderate_ × *Φ*_Moderate_	*Ω*_Low_ × *Φ*_Moderate_
	Low	*Ω*_High_ × *Φ*_Low_	*Ω*_Moderate_ × *Φ*_Low_	*Ω*_Low_ × *Φ*_Low_

**Table 4:** Construct risk strata using GM values [21].

*Φ*	*Ω* high	*Ω* moderate	*Ω* low
High	$R_{H,H}=\sqrt[{7}]{\overset{7}{\underset{i=1}{\prod }}\;\;\Omega_{\text{High}}\times \Phi_{\text{High}}}$	$R_{H,M}=\sqrt[{7}]{\overset{7}{\underset{i=1}{\prod }}\;\;\Omega_{\text{Moderate}}\times \Phi_{\text{High}}}$	$R_{H,L}=\sqrt[{7}]{\overset{7}{\underset{i=1}{\prod }}\;\;\Omega_{\text{Low}}\times \Phi_{\text{High}}}$
Moderate	$R_{M,H}=\sqrt[{7}]{\overset{7}{\underset{i=1}{\prod }}\;\;\Omega_{\text{High}}\times \Phi_{\text{Moderate}}}$	$R_{M,M}=\sqrt[{7}]{\overset{7}{\underset{i=1}{\prod }}\;\;\Omega_{\text{Moderate}}\times \Phi_{\text{Moderate}}}$	$R_{M,L}=\sqrt[{7}]{\overset{7}{\underset{i=1}{\prod }}\;\;\Omega_{\text{Low}}\times \Phi_{\text{Moderate}}}$
Low	$R_{L,H}=\sqrt[{7}]{\overset{7}{\underset{i=1}{\prod }}\;\;\Omega_{\text{High}}\times \Phi_{\text{Low}}}$	$R_{L,M}=\sqrt[{7}]{\overset{7}{\underset{i=1}{\prod }}\;\;\Omega_{\text{Moderate}}\times \Phi_{\text{Low}}}$	$R_{L,L}=\sqrt[{7}]{\overset{7}{\underset{i=1}{\prod }}\;\;\Omega_{\text{Low}}\times \Phi_{\text{Low}}}$

**Table 5:** The derived weights of sublevels of factors.

Factor	Risk level		
	High	Moderate	Low
T	0.5584	0.3446	0.0970
R	0.5905	0.2120	0.1975
D	0.4632	0.3905	0.1734
H	0.3694	0.3307	0.3000
G	0.5301	0.4480	0.0219
P	0.3694	0.3307	0.3000
U	0.5301	0.4480	0.0219
M	0.5905	0.2120	0.1975

**Table 6:** GM of different risk factor levels.

Cluster	High	Moderate	Low
High	0.2833	0.1017	0.0948
Moderate	0.2059	0.0739	0.0689
Low	0.0622	0.0223	0.0208

**Table 7:** Proposed risk clusters with their color scheme.

Risk cluster	GM	Color
Very high	0.2833-1.0000	Maroon
High	0.2059-0.2833	Red
Moderate	0.0689-0.2059	Yellow
Low	0.0208-0.0689	Light green
Very low	0.0000-0.0208	Green

**Table 8:** Ranks of districts in Sri Lanka and their dengue categories.

District	GM	Risk cluster	Relative dengue cases (%)
Ampara	0.0707	Moderate	0.53
Anuradhapura	0.0422	Low	1.66
Badulla	0.0488	Low	2.14
Batticaloa	0.0696	Moderate	3.20
Colombo	0.2230	High	19.57
Galle	0.0508	Low	3.58
Gampaha	0.1414	Moderate	18.07
Jaffna	0.0774	Moderate	3.47
Kalutara	0.1392	Moderate	6.26
Kandy	0.1414	Moderate	8.23
Kegalle	0.0488	Low	5.46
Kilinochchi	0.0793	Moderate	0.30
Kurunegala	0.1392	Moderate	6.43
Hambantota	0.0511	Low	2.04
Mannar	0.0711	Moderate	0.31
Matara	0.0462	Low	3.62
Mullaitivu	0.0700	Moderate	0.22
Nuwara Eliya	0.0462	Low	0.51
Puttalam	0.0488	Low	4.48
Ratnapura	0.0503	Low	6.45
Trincomalee	0.0619	Low	2.86
Vavuniya	0.0679	Low	0.61

## Data Availability

The datasets used during the current study are obtained from the Department of Census and Statistics, Epidemiology Unit of Health Ministry, Department of Meteorology, and Central Bank of Sri Lanka websites.

## Supplementary Materials

Supplementary Materials Supplementary material S1. Fuzzy pairwise comparison matrices. Supplementary material S2. Multiplicative interaction matrices. Supplementary material S3. MATLAB codes to find sensitivity of the weights. Supplementary material S4. R code to generate heat maps.
