# Supplementary material for: Fuzzy Multidimensional Model to Cluster Dengue Risk in Sri Lanka
Source: Biomed Res Int. 2020 Nov 4;2020:2420948. doi: 10.1155/2020/2420948 (PMC7661134; doi:10.1155/2020/2420948)
Supplement: Supplementary Materials — Supplementary material S1. Fuzzy pairwise comparison matrices. Supplementary material S2. Multiplicative interaction matrices. Supplementary material S3. MATLAB codes to find sensitivity of the weights. Supplementary material S4. R code to generate heat maps. [file 2420948.f1.pdf]

Supplementary Materials

S1 Fuzzy Pairwise Comparison Matrices

Table S1: Fuzzy pairwise comparison matrix of main factors when $\delta = 0.5$.

	**T**	**R**	**D**	**H**	**G**	**P**	**U**	**M**
**T**	(1,1,1)	(3/2,2,5/2)	(3/2,2,5/2)	(1,3/2,2)	(1/3,2/5,1/2)	(2/5,1/2,2/3)	(1/3,2/5,1/2)	(1/3,2/5,1/2)
**R**	(2/5,1/2,2/3)	(1,1,1)	(1/2,1,3/2)	(1,3/2,2)	(1/2,2/3,1)	(1/2,1,3/2)	(3/2,2,5/2)	(1/3,2/5,1/2)
**D**	(2/5,1/2,2/3)	(2/3,1,2)	(1,1,1)	(3/2,2,5/2)	(1/2,1,3/2)	(1,3/2,2)	(1/2,2/3,1)	(2/5,1/2,2/3)
**H**	(1/2,2/3,1)	(1/2,2/3,1)	(2/5,1/2,2/3)	(1,1,1)	(2/5,1/2,2/3)	(3/2,2,5/2)	(1/3,2/5,1/2)	(1/3,2/5,1/2)
**G**	(2,5/2,3)	(1,3/2,2)	(2/3,1,2)	(3/2,2,5/2)	(1,1,1)	(1,3/2,2)	(3/2,2,5/2)	(3/2,2,5/2)
**P**	(3/2,2,5/2)	(2/3,1,2)	(1/2,2/3,1)	(2/5,1/2,2/3)	(1/2,2/3,1)	(1,1,1)	(1/3,2/5,1/2)	(1/3,2/5,1/2)
**U**	(2,5/2,3)	(2/5,1/2,2/3)	(1,3/2,2)	(2,5/2,3)	(2/5,1/2,2/3)	(2,5/2,3)	(1,1,1)	(2/3,1,2)
**M**	(2,5/2,3)	(2,5/2,3)	(3/2,2,5/2)	(2,5/2,3)	(2/5,1/2,2/3)	(2,5/2,3)	(1/2,1,3/2)	(1,1,1)

Table S2: Fuzzy pairwise comparison matrix for the sub-factors of temperature when $\delta = 0.5$.

	**High**	**Moderate**	**Low**
**High**	(1,1,1)	(1,3/2,2)	(3/2,2,5/2)
**Moderate**	(1/2,2/3,1)	(1,1,1)	(1/2,1,3/2)
**Low**	(1/3,2/5,1/2)	(1/2,2/3,1)	(1,1,1)

Table S3: Fuzzy pairwise comparison matrix for the sub-factors of rainfall when $\delta = 0.5$.

	**High**	**Moderate**	**Low**
**High**	(1,1,1)	(1,3/2,2)	(2,5/2,3)
**Moderate**	(1/2,2/3,1)	(1,1,1)	(1,3/2,2)
**Low**	(2/5,1/2,2/3)	(2/3,1,2)	(1,1,1)

Table S4: Fuzzy pairwise comparison matrix for the sub-factors of number of rainy days when $\delta = 0.5$.

	**High**	**Moderate**	**Low**
**High**	(1,1,1)	(1/2,1,3/2)	(3/2,2,5/2)
**Moderate**	(2/3,1,2)	(1,1,1)	(1,3/2,2)
**Low**	(2/5,1/2,2/3)	(1/2,2/3,1)	(1,1,1)

Table S5: Fuzzy pairwise comparison matrix for the sub-factors of humidity when $\delta = 0.5$.

	**High**	**Moderate**	**Low**
**High**	(1,1,1)	(1/2,1,3/2)	(1,3/2,2)
**Moderate**	(2/3,1,2)	(1,1,1)	(1/2,1,3/2)
**Low**	(1/2,2/3,1)	(2/3,1,2)	(1,1,1)

Table S6: Fuzzy pairwise comparison matrix for the sub-factors of garbage when $\delta = 0.5$.

	**High**	**Moderate**	**Low**
**High**	(1,1,1)	(3/2,2,5/2)	(1,3/2,2)
**Moderate**	(2/5,1/2,2/3)	(1,1,1)	(2,5/2,3)
**Low**	(1/2,2/3,1)	(1/3,2/5,1/2)	(1,1,1)

Table S7: Fuzzy pairwise comparison matrix for the sub-factors of population density when $\delta = 0.5$.

	**High**	**Moderate**	**Low**
**High**	(1,1,1)	(1/2,1,3/2)	(1,3/2,2)
**Moderate**	(2/3,1,2)	(1,1,1)	(1/2,1,3/2)
**Low**	(1/2,2/3,1)	(2/3,1,2)	(1,1,1)

Table S8: Fuzzy pairwise comparison matrix for the sub-factors of urbanization when $\delta = 0.5$.

	**High**	**Moderate**	**Low**
**High**	(1,1,1)	(3/2,2,5/2)	(1,3/2,2)
**Moderate**	(2/5,1/2,2/3)	(1,1,1)	(2,5/2,3)
**Low**	(1/2,2/3,1)	(1/3,2/5,1/2)	(1,1,1)

Table S9: Fuzzy pairwise comparison matrix for the sub-factors of population movements when $\delta = 0.5$.

	**High**	**Moderate**	**Low**
**High**	(1,1,1)	(1,3/2,2)	(2,5/2,3)
**Moderate**	(1/2,2/3,1)	(1,1,1)	(1,3/2,2)
**Low**	(2/5,1/2,2/3)	(2/3,1,2)	(1,1,1)

S2 Multiplicative Interaction Matrices

Table S10: Multiplicative interaction matrix of temperature and most effective variable.

**Temperature**	**Population Movements**		
	**High**	**Moderate**	**Low**
High	0.3297352	0.1183808	0.1102840
Moderate	0.2034863	0.0730552	0.0680585
Low	0.0572785	0.0205640	0.0191575

Table S11: Multiplicative interaction matrix of rainfall and most effective variable.

**Rainfall**	**Population Movements**		
	**High**	**Moderate**	**Low**
High	0.3486903	0.125186	0.11662375
Moderate	0.1251860	0.044944	0.04187000
Low	0.1166238	0.041870	0.03900625

Table S12: Multiplicative interaction matrix of number of rainy days and most effective variable.

**Number of Rainy Days**	**Population Movements**		
	**High**	**Moderate**	**Low**
High	0.2735196	0.0981984	0.09148200
Moderate	0.2305903	0.0827860	0.07712375
Low	0.1023927	0.0367608	0.03424650

Table S13: Multiplicative interaction matrix of humidity and most effective variable.

**Humidity**	**Population Movements**		
	**High**	**Moderate**	**Low**
High	0.2181307	0.0783128	0.07295650
Moderate	0.1952784	0.0701084	0.06531325
Low	0.1771500	0.0636000	0.05925000

Table S14: Multiplicative interaction matrix of garbage and most effective variable.

**Garbage**	**Population Movements**		
	**High**	**Moderate**	**Low**
High	0.31302405	0.1123812	0.10469475
Moderate	0.26454400	0.0949760	0.08848000
Low	0.01293195	0.0046428	0.00432525

Table S15: Multiplicative interaction matrix of population density and most effective variable.

**Population Density**	**Population Movements**		
	**High**	**Moderate**	**Low**
High	0.2181307	0.0783128	0.07295650
Moderate	0.1952784	0.0701084	0.06531325
Low	0.1771500	0.0636000	0.05925000

Table S16: Multiplicative interaction matrix of urbanization and most effective variable.

**Urbanization**	**Population Movements**		
	**High**	**Moderate**	**Low**
High	0.31302405	0.1123812	0.10469475
Moderate	0.26454400	0.0949760	0.08848000
Low	0.01293195	0.0046428	0.00432525

S3 MATLAB Codes to Find Sensitivity of the Weights

S3.1 Code to Find Degrees of Possibilities

deg_posibility.m

```
function [V] = deg_posibility(l1,m1,u1,l2,m2,u2)
if (m1<=m2)
    V=1;
elseif (u2<=l1)
    V=0;
else
    V=(l1-u2)/((m2-u2)-(m1-l1));
end
end
```

S3.2 Code for Chang's Extent Analysis Method

Chang_hei.m

```
function [Normal_Weight] = Chang_hei(k)

if k==1
    l = [1,2-1,2-1,3/2-1,1/(5/2+1),1/(2+1),1/((5/2)+1),1/((5/2)+1);
         1/(2+1),1,1-0.5,3/2-1,1/((3/2)+1),1-0.5,2-1,1/((5/2)+1);
        1/(2+1),1/(1+1),1,2-1,1-0.5,3/2-1,1/((3/2)+1),1/(2+1);
        1/((3/2)+1),1/((3/2)+1),1/(2+1),1,1/(2+1),2-1,1/((5/2)+1)
        ,1/((5/2)+1); 5/2-1,5/2-1,1/(1+1),1/(2+1),1,3/2-1,2-1,2-1;
        2-1,1-1,1/((3/2)+1),1/(2+1),1/((3/2)+1),1,1/(5/2+1)
        ,1/(5/2+1); 5/2-1,1/(2+1),3/2-1,5/2-1,1/(2+1)
        ,5/2-1,1,1/(1+1); 5/2-1,5/2-1,2-1,5/2-1,1/(2+1)
        ,5/2-1,1-0.5,1];

    m = [1,2,2,3/2,2/5,1/2,2/5,2/5; 1/2,1,1,3/2,2/3,1,2,2/5;
        1/2,1,1,2,1,3/2,2/3,1/2; 2/3,2/3,1/2,1,1/2,2,2/5,2/5;
        5/2,3/2,1,2,1,3/2,2,2; 2,1,2/3,1/2,2/3,1,2/5,2/5;
        5/2,1/2,3/2,5/2,1/2,5/2,1,1; 5/2,5/2,2,5/2,1/2,5/2,1,1];

    u = [1,2+1,2+1,3/2+1,1/(5/2-1),1/(2-1),1/((5/2)-1),1/((5/2)-1);
         1/(2-1),1,1+1,3/2+1,1/((3/2)-1),1+1,2+1,1/((5/2)-1);
        1/(2-1),1/(1-1),1,2+1,1+1,3/2+1,1/((3/2)-1),1/(2-1);
        1/((3/2)-1),1/((3/2)-1),1/(2-1),1,1/(2-1),2+1,1/((5/2)-1)
        ,1/((5/2)-1);      5/2+1,5/2+1,(1+1),1/(2-1)
        ,1,3/2+1,2+1,2+1;2+1,1+1,1/((3/2)-1),1/(2-1),1/((3/2)-1)
        ,1,1/(5/2-1),1/(5/2-1);5/2+1,1/(2-1),3/2+1,5/2+1,1/(2-1)
        ,5/2+1,1,1+1; 5/2+1,5/2+1,2+1,5/2+1,1/(2-1),5/2+1,1+1,1];

else if k==1.5
        l = [1,2-1.4,2-1.4,3/2-1.4,1/(5/2+1.4),1/(2+1.4),1/((5/2)
            +1.4),1/((5/2)+1.4); 1/(2+1.4),1,1-1.4,3/2-1.4,1/((3/2)
            +1.4),1-1.4,2-1.4,1/((5/2)+1.4);1/(2+1.4),1/(1+1.4)
```

```
        ,1 ,2 −1.4 ,1 −1.4 ,3/2 −1.4 ,1/((3/2) +1.4) ,1/(2+1.4) ;
        1/((3/2) +1.4) ,1/((3/2) +1.4) ,1/(2+1.4) ,1 ,1/(2+1.4)
        ,2 −1.4 ,1/((5/2) +1.4) ,1/((5/2) +1.4)
        ;5/2 −1.4 ,5/2 −1.4 ,1/(1+1.4) ,1/(2+1.4)
        ,1 ,3/2 −1.4 ,2 −1.4 ,2 −1.4;2 −1.4 ,1 −1.4 ,1/((3/2) +1.4)
        ,1/(2+1.4) ,1/((3/2) +1.4) ,1 ,1/(5/2+1.4) ,1/(5/2+1.4) ;
        5/2 −1.4 ,1/(2+1.4) ,3/2 −1.4 ,5/2 −1.4 ,1/(2+1.4)
        ,5/2 −1.4 ,1 ,1/(1+1.4)
        ;5/2 −1.4 ,5/2 −1.4 ,2 −1.4 ,5/2 −1.4 ,1/(2+1.4)
        ,5/2 −1.4 ,1 −1.4 ,1];

    m = [1 ,2 ,2 ,3/2 ,2/5 ,1/2 ,2/5 ,2/5;  1/2 ,1 ,1 ,3/2 ,2/3 ,1 ,2 ,2/5;
        1/2 ,1 ,1 ,2 ,1 ,3/2 ,2/3 ,1/2;  2/3 ,2/3 ,1/2 ,1 ,1/2 ,2 ,2/5 ,2/5;
        5/2 ,3/2 ,1 ,2 ,1 ,3/2 ,2 ,2;  2 ,1 ,2/3 ,1/2 ,2/3 ,1 ,2/5 ,2/5;
        5/2 ,1/2 ,3/2 ,5/2 ,1/2 ,5/2 ,1 ,1;
        5/2 ,5/2 ,2 ,5/2 ,1/2 ,5/2 ,1 ,1];

    u = [1 ,2+1.4 ,2+1.4 ,3/2+1.4 ,1/(5/2 −1.4) ,1/(2 −1.4) ,1/((5/2)
        −1.4) ,1/((5/2) −1.4);  1/(2 −1.4) ,1 ,1+1.4 ,3/2+1.4 ,1/((3/2)
        −1.4) ,1+1.4 ,2+1.4 ,1/((5/2) −1.4) ;       1/(2 −1.4) ,1/(1 −1.4)
        ,1 ,2+1.4 ,1+1.4 ,3/2+1.4 ,1/((3/2) −1.4) ,1/(2 −1.4) ;
        1/((3/2) −1.4) ,1/((3/2) −1.4) ,1/(2 −1.4) ,1 ,1/(2 −1.4)
        ,2+1.4 ,1/((5/2) −1.4) ,1/((5/2) −1.4) ;
        5/2+1.4 ,5/2+1.4 ,1/(1 −1.4) ,1/(2 −1.4)
        ,1 ,3/2+1.4 ,2+1.4 ,2+1.4;  2+1.4 ,1+1.4 ,1/((3/2) −1.4)
        ,1/(2 −1.4) ,1/((3/2) −1.4) ,1 ,1/(5/2 −1.4) ,1/(5/2 −1.4) ;
        5/2+1.4 ,1/(2 −1.4) ,3/2+1.4 ,5/2+1.4 ,1/(2 −1.4)
        ,5/2+1.4 ,1 ,1/(1 −1.4) ;
        5/2+1.4 ,5/2+1.4 ,2+1.4 ,5/2+1.4 ,1/(2 −1.4)
        ,5/2+1.4 ,1+1.4 ,1];

else if k==1.1
    l = [1 ,2 −1 ,2 −1 ,3/2 −1 ,1/(5/2+1) ,1/(2+1) ,1/((5/2)+1) ,1/((5/2)
        +1);  1/(2+1) ,1 ,1 −0.5 ,3/2 −1 ,1/((3/2)+1)
        ,1 −0.5 ,2 −1 ,1/((5/2)+1);       1/(2+1) ,1/(1+1)
        ,1 ,2 −1 ,1 −0.5 ,3/2 −1 ,1/((3/2)+1) ,1/(2+1);  1/((3/2)+1)
        ,1/((3/2)+1) ,1/(2+1) ,1 ,1/(2+1) ,2 −1 ,1/((5/2)+1) ,1/((5/2)
        +1);       5/2 −1 ,5/2 −1 ,1/(1+1) ,1/(2+1) ,1 ,3/2 −1 ,2 −1 ,2 −1;
        2 −1 ,1 −1 ,1/((3/2)+1) ,1/(2+1) ,1/((3/2)+1) ,1 ,1/(5/2+1)
        ,1/(5/2+1) ;       5/2 −1 ,1/(2+1) ,3/2 −1 ,5/2 −1 ,1/(2+1)
        ,5/2 −1 ,1 ,1/(1+1) ;  5/2 −1 ,5/2 −1 ,2 −1 ,5/2 −1 ,1/(2+1)
        ,5/2 −1 ,1 −0.5 ,1];

    m = [1 ,2 ,2 ,3/2 ,2/5 ,1/2 ,2/5 ,2/5;  1/2 ,1 ,1 ,3/2 ,2/3 ,1 ,2 ,2/5;
        1/2 ,1 ,1 ,2 ,1 ,3/2 ,2/3 ,1/2;  2/3 ,2/3 ,1/2 ,1 ,1/2 ,2 ,2/5 ,2/5;
        5/2 ,3/2 ,1 ,2 ,1 ,3/2 ,2 ,2;  2 ,1 ,2/3 ,1/2 ,2/3 ,1 ,2/5 ,2/5;
        5/2 ,1/2 ,3/2 ,5/2 ,1/2 ,5/2 ,1 ,1;
        5/2 ,5/2 ,2 ,5/2 ,1/2 ,5/2 ,1 ,1];

    u = [1 ,2+1 ,2+1 ,3/2+1 ,1/(5/2 −1) ,1/(2 −1) ,1/((5/2) −1) ,1/((5/2)
        −1);  1/(2 −1) ,1 ,1+1 ,3/2+1 ,1/((3/2) −1) ,1+1 ,2+1 ,1/((5/2)
        −1);       1/(2 −1) ,1/(1 −1) ,1 ,2+1 ,1+1 ,3/2+1 ,1/((3/2) −1)
        ,1/(2 −1);  1/((3/2) −1) ,1/((3/2) −1) ,1/(2 −1) ,1 ,1/(2 −1)
        ,2+1 ,1/((5/2) −1) ,1/((5/2) −1);       5/2+1 ,5/2+1 ,(1+1)
        ,1/(2 −1) ,1 ,3/2+1 ,2+1 ,2+1;  2+1 ,1+1 ,1/((3/2) −1) ,1/(2 −1)
        ,1/((3/2) −1) ,1 ,1/(5/2 −1) ,1/(5/2 −1) ;       5/2+1 ,1/(2 −1)
        ,3/2+1 ,5/2+1 ,1/(2 −1) ,5/2+1 ,1 ,1+1;
```

```
        5/2+1,5/2+1,2+1,5/2+1,1/(2-1),5/2+1,1+1,1];

else if k==1.6
        l = [1,2-1.4,2-1.4,3/2-1.4,1/(5/2+1.4),1/(2+1.4),1/((5/2)
            +1.4),1/((5/2)+1.4);  1/(2+1.4),1,1-1.4,3/2-1.4,1/((3/2)
            +1.4),1-1.4,2-1.4,1/((5/2)+1.4);      1/(2+1.4),1/(1+1.4)
            ,1,2-1.4,1-1.4,3/2-1.4,1/((3/2)+1.4),1/(2+1.4);
            1/((3/2)+1.4),1/((3/2)+1.4),1/(2+1.4),1,1/(2+1.4)
            ,2-1.4,1/((5/2)+1.4),1/((5/2)+1.4);
            5/2-1.4,5/2-1.4,1/(1+1.4),1/(2+1.4)
            ,1,3/2-1.4,2-1.4,2-1.4;  2-1.4,1-1.4,1/((3/2)+1.4)
            ,1/(2+1.4),1/((3/2)+1.4),1,1/(5/2+1.4),1/(5/2+1.4);
            5/2-1.4,1/(2+1.4),3/2-1.4,5/2-1.4,1/(2+1.4)
            ,5/2-1.4,1,1/(1+1.4);
            5/2-1.4,5/2-1.4,2-1.4,5/2-1.4,1/(2+1.4)
            ,5/2-1.4,1-1.4,1];

        m = [1,2,2,3/2,2/5,1/2,2/5,2/5;  1/2,1,1,3/2,2/3,1,2,2/5;
            1/2,1,1,2,1,3/2,2/3,1/2;  2/3,2/3,1/2,1,1/2,2,2/5,2/5;
            5/2,3/2,1,2,1,3/2,2,2;  2,1,2/3,1/2,2/3,1,2/5,2/5;
            5/2,1/2,3/2,5/2,1/2,5/2,1,1;
            5/2,5/2,2,5/2,1/2,5/2,1,1];

        u = [1,2+1.4,2+1.4,3/2+1.4,1/(5/2-1.4),1/(2-1.4),1/((5/2)
            -1.4),1/((5/2)-1.4);  1/(2-1.4),1,1+1.4,3/2+1.4,1/((3/2)
            -1.4),1+1.4,2+1.4,1/((5/2)-1.4);      1/(2-1.4),1/(1-1.4)
            ,1,2+1.4,1+1.4,3/2+1.4,1/((3/2)-1.4),1/(2-1.4);
            1/((3/2)-1.4),1/((3/2)-1.4),1/(2-1.4),1,1/(2-1.4)
            ,2+1.4,1/((5/2)-1.4),1/((5/2)-1.4);
            5/2+1.4,5/2+1.4,1/(1-1.4),1/(2-1.4)
            ,1,3/2+1.4,2+1.4,2+1.4;  2+1.4,1+1.4,1/((3/2)-1.4)
            ,1/(2-1.4),1/((3/2)-1.4),1,1/(5/2-1.4),1/(5/2-1.4);
            5/2+1.4,1/(2-1.4),3/2+1.4,5/2+1.4,1/(2-1.4)
            ,5/2+1.4,1,1/(1-1.4);
            5/2+1.4,5/2+1.4,2+1.4,5/2+1.4,1/(2-1.4)
            ,5/2+1.4,1+1.4,1];


else if k==1.6
        l = [1,2-1.4,2-1.4,3/2-1.4,1/(5/2+1.4),1/(2+1.4),1/((5/2)
            +1.4),1/((5/2)+1.4);  1/(2+1.4),1,1-1.4,3/2-1.4,1/((3/2)
            +1.4),1-1.4,2-1.4,1/((5/2)+1.4);      1/(2+1.4),1/(1+1.4)
            ,1,2-1.4,1-1.4,3/2-1.4,1/((3/2)+1.4),1/(2+1.4);
            1/((3/2)+1.4),1/((3/2)+1.4),1/(2+1.4),1,1/(2+1.4)
            ,2-1.4,1/((5/2)+1.4),1/((5/2)+1.4);
            5/2-1.4,5/2-1.4,1/(1+1.4),1/(2+1.4)
            ,1,3/2-1.4,2-1.4,2-1.4;  2-1.4,1-1.4,1/((3/2)+1.4)
            ,1/(2+1.4),1/((3/2)+1.4),1,1/(5/2+1.4),1/(5/2+1.4);...
              5/2-1.4,1/(2+1.4),3/2-1.4,5/2-1.4,1/(2+1.4)
            ,5/2-1.4,1,1/(1+1.4);
            5/2-1.4,5/2-1.4,2-1.4,5/2-1.4,1/(2+1.4)
            ,5/2-1.4,1-1.4,1];

        m = [1,2,2,3/2,2/5,1/2,2/5,2/5;  1/2,1,1,3/2,2/3,1,2,2/5;
            1/2,1,1,2,1,3/2,2/3,1/2;  2/3,2/3,1/2,1,1/2,2,2/5,2/5;
            5/2,3/2,1,2,1,3/2,2,2;  2,1,2/3,1/2,2/3,1,2/5,2/5;
            5/2,1/2,3/2,5/2,1/2,5/2,1,1;
```

```
        5/2,5/2,2,5/2,1/2,5/2,1,1];

    u = [1,2+1.4,2+1.4,3/2+1.4,1/(5/2-1.4),1/(2-1.4),1/((5/2)
        -1.4),1/((5/2)-1.4);  1/(2-1.4),1,1+1.4,3/2+1.4,1/((3/2)
        -1.4),1+1.4,2+1.4,1/((5/2)-1.4);      1/(2-1.4),1/(1-1.4)
        ,1,2+1.4,1+1.4,3/2+1.4,1/((3/2)-1.4),1/(2-1.4);
        1/((3/2)-1.4),1/((3/2)-1.4),1/(2-1.4),1,1/(2-1.4)
        ,2+1.4,1/((5/2)-1.4),1/((5/2)-1.4);
        5/2+1.4,5/2+1.4,1/(1-1.4),1/(2-1.4)
        ,1,3/2+1.4,2+1.4,2+1.4;   2+1.4,1+1.4,1/((3/2)-1.4)
        ,1/(2-1.4),1/((3/2)-1.4),1,1/(5/2-1.4),1/(5/2-1.4);...
           5/2+1.4,1/(2-1.4),3/2+1.4,5/2+1.4,1/(2-1.4)
        ,5/2+1.4,1,1/(1-1.4);
        5/2+1.4,5/2+1.4,2+1.4,5/2+1.4,1/(2-1.4)
        ,5/2+1.4,1+1.4,1];

else if k==1.7
    l = [1,2-1.9,2-1.9,3/2-1.9,1/(5/2+1.9),1/(2+1.9),1/((5/2)
        +1.9),1/((5/2)+1.9);  1/(2+1.9),1,1-1.9,3/2-1.9,1/((3/2)
        +1.9),1-1.9,2-1.9,1/((5/2)+1.9);      1/(2+1.9),1/(1+1.9)
        ,1,2-1.9,1-1.9,3/2-1.9,1/((3/2)+1.9),1/(2+1.9);
        1/((3/2)+1.9),1/((3/2)+1.9),1/(2+1.9),1,1/(2+1.9)
        ,2-1.9,1/((5/2)+1.9),1/((5/2)+1.9);
        5/2-1.9,5/2-1.9,1/(1+1.9),1/(2+1.9)
        ,1,3/2-1.9,2-1.9,2-1.9;  2-1.9,1-1.9,1/((3/2)+1.9)
        ,1/(2+1.9),1/((3/2)+1.9),1,1/(5/2+1.9),1/(5/2+1.9);
        5/2-1.9,1/(2+1.9),3/2-1.9,5/2-1.9,1/(2+1.9)
        ,5/2-1.9,1,1/(1+1.9);
        5/2-1.9,5/2-1.9,2-1.9,5/2-1.9,1/(2+1.9)
        ,5/2-1.9,1-1.9,1];

    m = [1,2,2,3/2,2/5,1/2,2/5,2/5;  1/2,1,1,3/2,2/3,1,2,2/5;
        1/2,1,1,2,1,3/2,2/3,1/2;  2/3,2/3,1/2,1,1/2,2,2/5,2/5;
        5/2,3/2,1,2,1,3/2,2,2;  2,1,2/3,1/2,2/3,1,2/5,2/5;
        5/2,1/2,3/2,5/2,1/2,5/2,1,1;
        5/2,5/2,2,5/2,1/2,5/2,1,1];

    u = [1,2+1.9,2+1.9,3/2+1.9,1/(5/2-1.9),1/(2-1.9),1/((5/2)
        -1.9),1/((5/2)-1.9);  1/(2-1.9),1,1+1.9,3/2+1.9,1/((3/2)
        -1.9),1+1.9,2+1.9,1/((5/2)-1.9);  1/(2-1.9),1/(1-1.9)
        ,1,2+1.9,1+1.9,3/2+1.9,1/((3/2)-1.9),1/(2-1.9);
        1/((3/2)-1.9),1/((3/2)-1.9),1/(2-1.9),1,1/(2-1.9)
        ,2+1.9,1/((5/2)-1.9),1/((5/2)-1.9);
        5/2+1.9,5/2+1.9,1/(1-1.9),1/(2-1.9)
        ,1,3/2+1.9,2+1.9,2+1.9;  2+1.9,1+1.9,1/((3/2)-1.9)
        ,1/(2-1.9),1/((3/2)-1.9),1,1/(5/2-1.9),1/(5/2-1.9);
        5/2+1.9,1/(2-1.9),3/2+1.9,5/2+1.9,1/(2-1.9)
        ,5/2+1.9,1,1/(1-1.9);
        5/2+1.9,5/2+1.9,2+1.9,5/2+1.9,1/(2-1.9)
        ,5/2+1.9,1+1.9,1];

else

    l = [1,2-k,2-k,3/2-k,1/(5/2+k),1/(2+k),1/((5/2)+k),1/((5/2)
        +k);  1/(2+k),1,1-k,3/2-k,1/((3/2)+k),1-k,2-k,1/((5/2)+k
        );      1/(2+k),1/(1+k),1,2-k,1-k,3/2-k,1/((3/2)+k),1/(2+
        k);  1/((3/2)+k),1/((3/2)+k),1/(2+k),1,1/(2+k),2-k
```

```
            ,1/((5/2)+k),1/((5/2)+k);    5/2-k,5/2-k,1/(1+k),1/(2+k
            ),1,3/2-k,2-k,2-k; 2-k,1-k,1/((3/2)+k),1/(2+k),1/((3/2)
            +k),1,1/(5/2+k),1/(5/2+k);    5/2-k,1/(2+k),3/2-k,5/2-k
            ,1/(2+k),5/2-k,1,1/(1+k); 5/2-k,5/2-k,2-k,5/2-k,1/(2+k)
            ,5/2-k,1-k,1];

        m = [1,2,2,3/2,2/5,1/2,2/5,2/5; 1/2,1,1,3/2,2/3,1,2,2/5;
            1/2,1,1,2,1,3/2,2/3,1/2; 2/3,2/3,1/2,1,1/2,2,2/5,2/5;
            5/2,3/2,1,2,1,3/2,2,2; 2,1,2/3,1/2,2/3,1,2/5,2/5;
            5/2,1/2,3/2,5/2,1/2,5/2,1,1;
            5/2,5/2,2,5/2,1/2,5/2,1,1];

        u = [1,2+k,2+k,3/2+k,1/(5/2-k),1/(2-k),1/((5/2)-k),1/((5/2)
            -k); 1/(2-k),1,1+k,3/2+k,1/((3/2)-k),1+k,2+k,1/((5/2)-k
            );    1/(2-k),1/(1-k),1,2+k,1+k,3/2+k,1/((3/2)-k),1/(2-
            k); 1/((3/2)-k),1/((3/2)-k),1/(2-k),1,1/(2-k),2+k
            ,1/((5/2)-k),1/((5/2)-k);    5/2+k,5/2+k,1/(1-k),1/(2-k
            ),1,3/2+k,2+k,2+k; 2+k,1+k,1/((3/2)-k),1/(2-k),1/((3/2)
            -k),1,1/(5/2-k),1/(5/2-k);    5/2+k,1/(2-k),3/2+k,5/2+k
            ,1/(2-k),5/2+k,1,1/(1-k); 5/2+k,5/2+k,2+k,5/2+k,1/(2-k)
            ,5/2+k,1+k,1];

end
end
end
end
end
end

%% Calculate  [Sigma Sigma M]-1
l_sum =sum(l(1,:))+sum(l(2,:))+sum(l(3,:))+sum(l(4,:))+sum(l(5,:))+
    sum(l(6,:))+ sum(l(7,:))+ sum(l(8,:));

m_sum =sum(m(1,:))+sum(m(2,:))+sum(m(3,:))+sum(m(4,:))+sum(m(5,:))+
    sum(m(6,:))+ sum(m(7,:))+ sum(m(8,:));;

u_sum =sum(u(1,:))+sum(u(2,:))+sum(u(3,:))+sum(u(4,:))+sum(u(5,:))+
    sum(u(6,:))+ sum(u(7,:))+ sum(u(8,:));;

sum_trans = [1/u_sum , 1/m_sum , 1/l_sum];

%% Row Sum
T = [sum(l(1,:)), sum(m(1,:)), sum(u(1,:))];
R = [sum(l(2,:)), sum(m(2,:)), sum(u(2,:))];
D = [sum(l(3,:)), sum(m(3,:)), sum(u(3,:))];
H = [sum(l(4,:)), sum(m(4,:)), sum(u(4,:))];
G = [sum(l(5,:)), sum(m(5,:)), sum(u(5,:))];
P = [sum(l(6,:)), sum(m(6,:)), sum(u(6,:))];
U = [sum(l(7,:)), sum(m(7,:)), sum(u(7,:))];
M = [sum(l(8,:)), sum(m(8,:)), sum(u(8,:))];

%% Calculate Sigma M * [Sigma Sigma M]-1
S_T= T.* sum_trans
S_R= R.* sum_trans
S_D= D.* sum_trans
S_H= H.* sum_trans
S_G= G.* sum_trans
```

```
S_P= P.* sum_trans
S_U= U.* sum_trans
S_M= M.* sum_trans

%% Degree of Possibility
V_T_great_R=deg_posibility(S_R(1,1),S_R(1,2),S_R(1,3),S_T(1,1),S_T
    (1,2),S_T(1,3))
V_T_great_D=deg_posibility(S_D(1,1),S_D(1,2),S_D(1,3),S_T(1,1),S_T
    (1,2),S_T(1,3))
V_T_great_H=deg_posibility(S_H(1,1),S_H(1,2),S_H(1,3),S_T(1,1),S_T
    (1,2),S_T(1,3))
V_T_great_G=deg_posibility(S_G(1,1),S_G(1,2),S_G(1,3),S_T(1,1),S_T
    (1,2),S_T(1,3))
V_T_great_P=deg_posibility(S_P(1,1),S_P(1,2),S_P(1,3),S_T(1,1),S_T
    (1,2),S_T(1,3))
V_T_great_U=deg_posibility(S_U(1,1),S_U(1,2),S_U(1,3),S_T(1,1),S_T
    (1,2),S_T(1,3))
V_T_great_M=deg_posibility(S_M(1,1),S_M(1,2),S_M(1,3),S_T(1,1),S_T
    (1,2),S_T(1,3))

V_R_great_T=deg_posibility(S_T(1,1),S_T(1,2),S_T(1,3),S_R(1,1),S_R
    (1,2),S_R(1,3))
V_R_great_D=deg_posibility(S_D(1,1),S_D(1,2),S_D(1,3),S_R(1,1),S_R
    (1,2),S_R(1,3))
V_R_great_H=deg_posibility(S_H(1,1),S_H(1,2),S_H(1,3),S_R(1,1),S_R
    (1,2),S_R(1,3))
V_R_great_G=deg_posibility(S_G(1,1),S_G(1,2),S_G(1,3),S_R(1,1),S_R
    (1,2),S_R(1,3))
V_R_great_P=deg_posibility(S_P(1,1),S_P(1,2),S_P(1,3),S_R(1,1),S_R
    (1,2),S_R(1,3))
V_R_great_U=deg_posibility(S_U(1,1),S_U(1,2),S_U(1,3),S_R(1,1),S_R
    (1,2),S_R(1,3))
V_R_great_M=deg_posibility(S_M(1,1),S_M(1,2),S_M(1,3),S_R(1,1),S_R
    (1,2),S_R(1,3))


V_D_great_T=deg_posibility(S_T(1,1),S_T(1,2),S_T(1,3),S_D(1,1),S_D
    (1,2),S_D(1,3))
V_D_great_R=deg_posibility(S_R(1,1),S_R(1,2),S_R(1,3),S_D(1,1),S_D
    (1,2),S_D(1,3))
V_D_great_H=deg_posibility(S_H(1,1),S_H(1,2),S_H(1,3),S_D(1,1),S_D
    (1,2),S_D(1,3))
V_D_great_G=deg_posibility(S_G(1,1),S_G(1,2),S_G(1,3),S_D(1,1),S_D
    (1,2),S_D(1,3))
V_D_great_P=deg_posibility(S_P(1,1),S_P(1,2),S_P(1,3),S_D(1,1),S_D
    (1,2),S_D(1,3))
V_D_great_U=deg_posibility(S_U(1,1),S_U(1,2),S_U(1,3),S_D(1,1),S_D
    (1,2),S_D(1,3))
V_D_great_M=deg_posibility(S_M(1,1),S_M(1,2),S_M(1,3),S_D(1,1),S_D
    (1,2),S_D(1,3))

V_H_great_T=deg_posibility(S_T(1,1),S_T(1,2),S_T(1,3),S_H(1,1),S_H
    (1,2),S_H(1,3))
V_H_great_R=deg_posibility(S_R(1,1),S_R(1,2),S_R(1,3),S_H(1,1),S_H
    (1,2),S_H(1,3))
V_H_great_D=deg_posibility(S_D(1,1),S_D(1,2),S_D(1,3),S_H(1,1),S_H
    (1,2),S_H(1,3))
```

```
V_H_great_G=deg_posibility(S_G(1,1),S_G(1,2),S_G(1,3),S_H(1,1),S_H
    (1,2),S_H(1,3))
V_H_great_P=deg_posibility(S_P(1,1),S_P(1,2),S_P(1,3),S_H(1,1),S_H
    (1,2),S_H(1,3))
V_H_great_U=deg_posibility(S_U(1,1),S_U(1,2),S_U(1,3),S_H(1,1),S_H
    (1,2),S_H(1,3))
V_H_great_M=deg_posibility(S_M(1,1),S_M(1,2),S_M(1,3),S_H(1,1),S_H
    (1,2),S_H(1,3))

V_G_great_T=deg_posibility(S_T(1,1),S_T(1,2),S_T(1,3),S_G(1,1),S_G
    (1,2),S_G(1,3))
V_G_great_R=deg_posibility(S_R(1,1),S_R(1,2),S_R(1,3),S_G(1,1),S_G
    (1,2),S_G(1,3))
V_G_great_D=deg_posibility(S_D(1,1),S_D(1,2),S_D(1,3),S_G(1,1),S_G
    (1,2),S_G(1,3))
V_G_great_H=deg_posibility(S_H(1,1),S_H(1,2),S_H(1,3),S_G(1,1),S_G
    (1,2),S_G(1,3))
V_G_great_P=deg_posibility(S_P(1,1),S_P(1,2),S_P(1,3),S_G(1,1),S_G
    (1,2),S_G(1,3))
V_G_great_U=deg_posibility(S_U(1,1),S_U(1,2),S_U(1,3),S_G(1,1),S_G
    (1,2),S_G(1,3))
V_G_great_M=deg_posibility(S_M(1,1),S_M(1,2),S_M(1,3),S_G(1,1),S_G
    (1,2),S_G(1,3))

V_P_great_T=deg_posibility(S_T(1,1),S_T(1,2),S_T(1,3),S_P(1,1),S_P
    (1,2),S_P(1,3))
V_P_great_R=deg_posibility(S_R(1,1),S_R(1,2),S_R(1,3),S_P(1,1),S_P
    (1,2),S_P(1,3))
V_P_great_D=deg_posibility(S_D(1,1),S_D(1,2),S_D(1,3),S_P(1,1),S_P
    (1,2),S_P(1,3))
V_P_great_H=deg_posibility(S_H(1,1),S_H(1,2),S_H(1,3),S_P(1,1),S_P
    (1,2),S_P(1,3))
V_P_great_G=deg_posibility(S_G(1,1),S_G(1,2),S_G(1,3),S_P(1,1),S_P
    (1,2),S_P(1,3))
V_P_great_U=deg_posibility(S_U(1,1),S_U(1,2),S_U(1,3),S_P(1,1),S_P
    (1,2),S_P(1,3))
V_P_great_M=deg_posibility(S_M(1,1),S_M(1,2),S_M(1,3),S_P(1,1),S_P
    (1,2),S_P(1,3))

V_U_great_T=deg_posibility(S_T(1,1),S_T(1,2),S_T(1,3),S_U(1,1),S_U
    (1,2),S_U(1,3))
V_U_great_R=deg_posibility(S_R(1,1),S_R(1,2),S_R(1,3),S_U(1,1),S_U
    (1,2),S_U(1,3))
V_U_great_D=deg_posibility(S_D(1,1),S_D(1,2),S_D(1,3),S_U(1,1),S_U
    (1,2),S_U(1,3))
V_U_great_H=deg_posibility(S_H(1,1),S_H(1,2),S_H(1,3),S_U(1,1),S_U
    (1,2),S_U(1,3))
V_U_great_G=deg_posibility(S_G(1,1),S_G(1,2),S_G(1,3),S_U(1,1),S_U
    (1,2),S_U(1,3))
V_U_great_P=deg_posibility(S_P(1,1),S_P(1,2),S_P(1,3),S_U(1,1),S_U
    (1,2),S_U(1,3))
V_U_great_M=deg_posibility(S_M(1,1),S_M(1,2),S_M(1,3),S_U(1,1),S_U
    (1,2),S_U(1,3))

V_M_great_T=deg_posibility(S_T(1,1),S_T(1,2),S_T(1,3),S_M(1,1),S_M
    (1,2),S_M(1,3))
V_M_great_R=deg_posibility(S_R(1,1),S_R(1,2),S_R(1,3),S_M(1,1),S_M
```

```
    (1,2),S_M(1,3))
V_M_great_D=deg_posibility(S_D(1,1),S_D(1,2),S_D(1,3),S_M(1,1),S_M
    (1,2),S_M(1,3))
V_M_great_H=deg_posibility(S_H(1,1),S_H(1,2),S_H(1,3),S_M(1,1),S_M
    (1,2),S_M(1,3))
V_M_great_G=deg_posibility(S_G(1,1),S_G(1,2),S_G(1,3),S_M(1,1),S_M
    (1,2),S_M(1,3))
V_M_great_P=deg_posibility(S_P(1,1),S_P(1,2),S_P(1,3),S_M(1,1),S_M
    (1,2),S_M(1,3))
V_M_great_U=deg_posibility(S_U(1,1),S_U(1,2),S_U(1,3),S_M(1,1),S_M
    (1,2),S_M(1,3))

%% Minimizing and Normalizing
Weight = [min([V_T_great_R,V_T_great_H,V_T_great_G,V_T_great_P,
    V_T_great_U,V_T_great_M]) ,...
    min([V_R_great_T,V_R_great_D,V_R_great_H,V_R_great_G,
        V_R_great_P,V_R_great_U,V_R_great_M]) ,...
    min([V_D_great_T,V_D_great_R,V_D_great_H,V_D_great_G,
        V_D_great_P,V_D_great_U,V_D_great_M]) ,...
    min([V_H_great_T,V_H_great_R,V_H_great_D,V_H_great_G,
        V_H_great_P,V_H_great_U,V_H_great_M]) ,...
    min([V_G_great_T,V_G_great_R,V_G_great_D,V_G_great_H,
        V_G_great_P,V_G_great_U,V_G_great_M]) ,...
    min([V_P_great_T,V_P_great_R,V_P_great_D,V_P_great_H,
        V_P_great_G,V_P_great_U,V_P_great_M]) ,...
    min([V_U_great_T,V_U_great_R,V_U_great_D,V_U_great_H,
        V_U_great_G,V_U_great_P,V_U_great_M]) ,...
    min([V_M_great_T,V_M_great_R,V_M_great_D,V_M_great_H,
        V_M_great_G,V_M_great_P,V_M_great_U])]

Normal_Weight = Weight./sum(Weight)
```

S3.3 Code to Plot Weights

Main Program

```
clc; clear all;
weights = 0:0.1:2;


for i=1:21
    prop_wei_h1  = Chang_hei1(weights(i));
    T(i) = prop_wei_h1(1);
    R(i) = prop_wei_h1(2);
    D(i) = prop_wei_h1(3);
    H(i) = prop_wei_h1(4);
    G(i) = prop_wei_h1(5);
    P(i) = prop_wei_h1(6);
    U(i) = prop_wei_h1(7);
    M(i) = prop_wei_h1(8);
end

%% Draw Figure
figure()
plot(weights,T,'LineWidth',2.5,'Color','black')
```

```
hold on
plot(weights,R,'LineWidth',2.5,'Color','red')
plot(weights,D,'LineWidth',2.5,'Color','magenta')
plot(weights,H,'LineWidth',2.5,'Color','blue')
plot(weights,G,'LineWidth',2.5,'Color','green')
plot(weights,P,'LineWidth',2.5,'Color',[0, 0.5, 0])
plot(weights,U,'LineWidth',2.5,'Color',[0.4940, 0.1840, 0.5560])
plot(weights,M,'LineWidth',2.5,'Color',[0.8500, 0.3250, 0.0980])


xlabel('\delta','fontsize',14,'FontName','Timesnewroman')
ylabel('{Weight}','fontsize',14,'FontName','Timesnewroman')
legend({'T','R','D','H','G','P','U','M'},'FontSize',14,'FontWeight'
    ,'bold')
set(gca,'fontsize',14)
hold off
```

S4 R Code to Generate Heat Maps

```
data <- read.csv("data.csv", check.names=FALSE)
rnames <- data[,1]
mat_data <- data.matrix(data[,2:ncol(data)])  # transform columns
    into a matrix
rownames(mat_data) <- rnames                  # assign row names

#########################################################
### Customizing and plotting the heat map
#########################################################

# creates a own color palette from red to green
my_palette <- c("yellowgreen","yellow2","red")# (n = 599)


par(xpd = T, mar = par()$mar + c(0,0,0,7))
col_breaks  =c(0, 50, 100, 500)# defines the color breaks manually

heatmap.2(mat_data, cellnote = mat_data, breaks = col_breaks,
col = my_palette,dendrogram=c("none"),
 key=F, trace="none",notecol="black",cexRow=0.8,cexCol=1.3,
xlab="Year",ylab='', main = '',srtCol=360,margin=c(5, 10),Rowv=
    FALSE, Colv=FALSE)

regions <- c("High","Moderate","Low")
colors <- c("red","yellow2","yellowgreen")
legend(0.3,0.9, regions, bty='L', fill = colors, horiz=TRUE, inset=
    c(-0.6,14),cex=0.65)
```
